# No Association between *FC****γ****R3B* Copy Number Variation and Susceptibility to Biopsy-Proven Giant Cell Arteritis

**DOI:** 10.1155/2013/514914

**Published:** 2013-08-20

**Authors:** Emma Dunstan, Sue Lester, Rachel Black, Maureen Rischmueller, Helen Chan, Alex W. Hewitt, Catherine L. Hill

**Affiliations:** ^1^Rheumatology Department, The Queen Elizabeth Hospital, Woodville South, SA 5011, Australia; ^2^The Health Observatory, Discipline of Medicine, The University of Adelaide, Adelaide, SA 5005, Australia; ^3^Discipline of Medicine, The University of Adelaide, Adelaide, SA 5005, Australia; ^4^Centre for Eye Research, Royal Victorian Eye and Ear Hospital, University of Melbourne, East Melbourne, VIC 3002, Australia; ^5^Lions Institute, University of Western Australia, Nedlands, WA 6009, Australia

## Abstract

*Objective*. To determine the relationship between FCGR3B gene copy number variation (CNV) and biopsy proven giant cell arteritis (GCA). *Methods*. *FCGR3B* CNV was determined in 139 Australian biopsy proven GCA patients and 162 population matched controls, using a duplex qPCR assay and RNase P as the reference gene. Copy number was determined using Copy Caller software (v.1.0, Applied Biosystems, USA). CNV genotypes were classified into 3 groups (<2, 2, 3+) for analysis purposes, and analysis was performed using logistic regression. *Results*. All GCA patients had a positive temporal artery biopsy, and the most common presenting symptoms were visual disturbance and temporal headache. The mean age of patients at biopsy was 74 years (range 51–94) and 88/139 (63%) were female. The frequency of low (<2) *FCGR3B* copy number was comparable between GCA patients (9/139 = 6.5%) and controls (10/162 = 6.2%), as was the frequency of high (3+) *FCGR3B* copy number (15/130 (10.8%) in GCA patients versus 13/162 (8.0%) in controls). Overall there was no evidence that *FCGR3B* CNV frequencies differed between GCA patients and controls (*χ* ^2^ = 0.75, *df* = 2, *P* = 0.69). *Conclusion*. *FCGR3B* CNV is not associated with GCA; however, replicate studies are required.

## 1. Introduction

Giant cell arteritis (GCA), also known as temporal arteritis, is a systemic inflammatory vasculitis which primarily affects medium to large extracranial arteries of the head and neck and can result in stroke and blindness. GCA typically affects people aged over 50 years and incidence rates increase with advancing age, peaking around 80 years of age [1]. GCA is 2-3 times more likely to affect females and is more commonly diagnosed in Caucasians than in any other ethnic background with the highest incidence observed in populations of Scandinavian descent [2]. 

The pathogenesis of GCA is not understood, although environmental, infectious, and genetic risk factors have been implicated. Familial aggregation and established associations with *HLA-DR4* provide evidence for a genetic component to GCA [3–5]. Multiple genetic association studies have been performed on a number of immune response genes. However, the majority of these studies have been performed on a single GCA cohort from north-western Spain and, to date, have failed to confirm any additional genetic associations.

One gene of interest is *Fc gamma receptor 3B* (*FCGR3B*) which exhibits gene copy number variation (CNV), an important source of quantitative genetic variation. Copy number variation is a departure from the normal diploid number of genes (*n* = 2) which may arise through gene duplication and deletion events. An increasing number of CNVs have been characterised in the human genome with implications for both evolution and disease susceptibility [6]. CNV has been well characterised in the *FCGR* gene cluster on chromosome 1q23. This cluster carries five highly homologous genes that encode for low-affinity receptors for IgG-complexed antigens, which are expressed widely throughout the haematopoietic system. These low-affinity Fc-gamma receptors are involved in the regulation of a multitude of innate and adaptive immune responses, with implications for both response to infection and susceptibility to autoimmunity [7]. 

CNV in the *FCGR3B* gene is of particular interest. FCGR3B is expressed almost exclusively on neutrophils [7], and there is a clear correlation between gene copy number and FCGR3B cell surface expression, neutrophil adherence to IgG-coated surfaces, and immune complex uptake [8]. Further, multiple studies have identified low *FCGR3B* CN (i.e., <2 copies) as a risk factor for systemic autoimmune diseases, such as systemic lupus erythematosus [9–11], rheumatoid arthritis [12, 13], primary Sjögren's syndrome [10, 11], and scleroderma [14], which is interpreted in terms of the important role that Fc receptors play in the clearance of immune complexes. In contrast, given the importance of neutrophil activation in vascular inflammation [15], it is plausible that high *FCGR3B* copy number may in fact predispose to receptor-mediated neutrophil activation and therefore vasculitis.

Previous studies in relation to *FCGR3B* CNV and vasculitis are inconclusive. Associations have been reported with both low [9] and high [8] copy number, whilst other studies have not identified such an association (reviewed in [16]). We have also previously reported that *FCGR3B* CNV is not a risk factor for Behcet's disease [17] in Iranian patients. The aim of this study was to evaluate the association between *FCGR3B* CNV and biopsy-proven GCA in an Australian patient cohort.

## 2. Materials and Methods

### 2.1. Subjects

One hundred and thirty-nine Australian biopsy-proven GCA patients were recruited through the South Australian Giant Cell Arteritis Registry and the Royal Victorian Eye and Ear Hospital. This study has ethics approval from the Queen Elizabeth Hospital, Royal Adelaide Hospital, Repatriation General Hospital and Flinders Medical Centre in South Australia, and the Royal Victorian Eye and Ear Hospital in Victoria, and all participants provided written, informed consent. A total of 162 population controls (53% female, median age 56 years) were used for comparison. 

### 2.2. FCGR3B Copy Number Typing

Genomic *FCGR3B* copy number was assessed using a custom TaqMan quantitative real-time PCR (qPCR) method, as previously described [11, 12, 17]. Briefly, a duplex TaqMan copy number assay was performed, using FCGR3B-specific primers (Applied Biosystems, Hs04211858, FAM-MGB dual-labeled probe) and RNase P (Applied Biosystems, product 4403326, VIC-TAMRA dual-labeled probe) as the reference assay. The assay was performed according to the manufacturer's instructions, and PCR reactions were run on an Applied Biosystems 7300 Real-Time PCR machine. All samples were tested in triplicate, and fluorescence signals were normalised to ROX. Copy number was determined using Copy Caller software (v.1.0, Applied Biosystems, USA), and results were accepted only when calling confidence was >80% and ΔCq standard deviation between replicates was <0.20.

### 2.3. Statistical Analysis

Analysis of *FCGR3B* CNV was performed using logistic regression. Effect sizes were reported as odds ratios (OR) with 95% confidence intervals (95% CI). 

## 3. Results

The demographic characteristics of the GCA patients are displayed in Table 1. All patients had a positive temporal artery biopsy. The mean age of patients at biopsy was 74 years (range 51–94) and 88/139 (63%) were female. 

Five different copy number variant genotypes were observed in this study, corresponding to 0, 1, 2, 3, 4 *FCGR3B *copies in a diploid individual. Because 0 and 4 copies were infrequent, genotypes were classified into 3 groups (< 2, 2, 3+) for analysis purposes. The most common *FCGR3B* gene copy number was 2 (normal diploid number) which was identified in 139/162 (86%) of controls and 114/138 (83%) of GCA patients (Table 2). There was no evidence that the overall distribution of *FCGR3B* CNV genotypes differed significantly between GCA patients and controls (global *χ* ^2^ = 0.75, *df* = 2, *P* = 0.69) nor was there any evidence of a specific difference between GCA patient and controls for low (<2, *P* = 0.85) or high (3+, *P* = 0.39) *FCGR3B* copy number (Table 2). 

## 4. Discussion

Whilst an association between *FCGR3B* low copy number (<2) and susceptibility to systemic autoimmune diseases is well established, with potential mechanisms relating to receptor clearance of immune complexes, and perhaps the most plausible hypothesis in relation to vasculitis is that high FCGR3B copy number may predispose via increased receptor-mediated neutrophil activation. This is the first study to examine the relationship between *FCGR3B* CNV and susceptibility to biopsy-proven GCA, and we report no evidence of an association with either high or low copy number.

Previous studies have reported intriguing, but conflicting, relationships between *FCGR3B* copy number and vasculitis in the context of different diseases. Both low and high *FCGR3B* copy number (<2) have been associated with anti-neutrophil cytoplasmic antibody-associated systemic vasculitides [8, 9], with a third study [18] observing no association. Other studies have reported no *FCGR3B* CNV associations with Kawasaki disease [19], antiglomerular basement membrane disease [20], and Behcet's disease [17] nor with vasculitis complicating systemic lupus erythematosus [21].

The *FCGR* gene cluster is a complex genomic region, with both SNP and CNV polymorphism. While we were unable to demonstrate an association between *FCGR3B* copy number and GCA in this study, there are putative links to polymorphism in this region with systemic vasculitides, as SNPs within the *FCGR* gene cluster have been associated with GCA, Behcet's diseases and Kawasaki's disease [22, 23]. However, the high degree of sequence homology between the segmental duplications in the *FCGR* cluster has hindered sequence annotation and unambiguous SNP mapping in this region, and therefore the interpretation and replication of these studies are unclear.

The strength of our study is the selection of patients with biopsy confirmation of GCA, allowing accurate ascertainment of cases. A limitation of our study is its relatively small sample size. The relatively wide effect size confidence intervals indicate that an association between GCA and either low or high *FCGR3B* copy number cannot be definitively excluded on the results of this study alone, and future replication studies are required. In general, genetic association studies with GCA have been hindered by the difficulties in collection of DNA samples from elderly patients in an essentially rare, late onset disease. Previously published genetic studies for GCA all have similarly small patient samples sizes, and indeed there is a paucity of different GCA patient cohorts for this type of research. International collaboration will be essential to collect large patient datasets and samples, with prospective recruitment at the time of diagnosis optimal for capturing appropriate samples with accompanying clinical and laboratory data.

## 5. Conclusion

The results of this study indicate that *FCGR3B* copy number variation is not a risk factor for GCA. Larger replication studies will be required to definitively establish any relationship between *FCGR3B* CNV and GCA and indeed other vasculitides.

## Figures and Tables

**Table 1:** Demographic features and presenting symptoms of patients with giant cell arteritis. Clinical data at presentation was not available for all patients.

Age at diagnosis, median (range)	74 years (51–94)
Gender, % female	88/139 (63%)
Temporal artery biopsy positive	139/139 (100%)
Visual disturbance	57/78 (73%)
Temporal headache	70/77 (65%)
Jaw claudication	46/78 (58%)
Scalp tenderness	37/78 (47%)
Loss of vision	21/71 (30%)

**Table 2:** FCGR3B gene copy number distribution in GCA patients and controls. Odds ratios (OR) and *P* values were derived by logistic regression, with the normal diploid copy number (2) selected as the reference category.

FCGR3B copy number	Control *N* = 162 (%)	GCA *N* = 138 (%)	OR (95% CI)	*P* Value
<2	10 (6.2)	9 (6.5)	1.1 (0.4, 2.8)	0.85
2	139 (85.8)	114 (82.6)	1	—
3+	13 (8.0)	15 (10.8)	1.4 (0.6, 3.1)	0.39

Chi-squared = 0.75, degrees freedom = 2, and *P* value = 0.69.
